# Lung retransplantation during the Lung Allocation Score era: Outcomes from a large single center

**DOI:** 10.1016/j.jhlto.2026.100501

**Published:** 2026-01-30

**Authors:** Kemarut Laothamatas, Cristiana Salvatori, Harpreet Singh Grewal, Mark Sonnick, Elena-Rodica Vasilescu, Lori Shah, Hilary Robbins, Angela DiMango, Gabriela Magda, Bryan P. Stanifer, Joshua R. Sonett, Frank D’Ovidio, Philippe Lemaitre, Luke Benvenuto, Selim M. Arcasoy

**Affiliations:** a Lung Transplant Program, Columbia University Irving Medical Center and New York-Presbyterian Hospital, New York, NY; b Division of Pulmonary and Critical Care Medicine, Brigham and Women’s Hospital, Boston, MA; c Lung Transplant Program, Transplant Institute, NYU Langone Health, New York, NY; d Department of Pathology and Cell Biology, Columbia University Irving Medical Center and New York-Presbyterian Hospital, New York, NY; e Section of Thoracic Surgery, Department of Surgery, Columbia University Irving Medical Center, New York, NY

**Keywords:** Lung transplantation, Retransplantation, Lung Allocation Score (LAS), Mortality outcomes, Chronic lung allograft dysfunction (CLAD)

## Abstract

**Background:**

Lung retransplantation (ReTx) is the only definitive treatment for advanced chronic lung allograft dysfunction (CLAD). ReTx outcomes are historically inferior to those of primary transplant. Identification of clinically significant risk factors could guide candidate selection and resource allocation to optimize outcomes.

**Methods:**

We conducted a single-center, retrospective cohort study of all lung transplants performed at Columbia University Medical Center (CUMC) from 2005 to 2023. The primary outcomes of survival and freedom from CLAD after ReTx were analyzed using Cox regression models.

**Results:**

We identified 51 lung retransplant recipients and compared them with 1149 primary lung transplant recipients. ReTx accounted for 3.9% of all transplants, and almost all were for CLAD-BOS.

One-, three-, five-, and ten-year survival after ReTx was 96%, 72%, 55%, and 28%, respectively (median = 5.61 [3.30–8.51] years). Characteristics associated with worse survival following ReTx included age ≥ 55 (adjusted HR 2.6; 95% CI, 1.39–5.02) and early-onset CLAD following ReTx (HR 4.86; 95% CI, 1.94–12.2). Pretransplant mechanical ventilation or ECMO support, type of surgical procedure, and the interval between primary and re-transplantation were not significantly associated with survival. Overall, our center’s outcomes after lung ReTx were more favorable than those previously reported, likely due to more restrictive candidate selection. These findings highlight the importance of careful patient selection. Multicenter prospective studies are needed to further refine risk stratification and optimize ReTx outcomes.

## Introduction

The number of lung transplants performed worldwide has been steadily rising. Over 3300 lung transplants were performed in the United States in 2024, a 10.4% increase from 2023 which marks the largest proportional growth of all solid organ transplants.^1^ While short-term post-lung transplant survival is excellent, long-term survival remains suboptimal with 5-year survival of 60%.^2^

Currently, the most significant barrier to long-term survival in lung transplant patients remains chronic lung allograft dysfunction (CLAD) for which the only definitive treatment is lung re-transplantation (ReTx). Existing literature shows that ReTx is associated with significantly lower survival than primary lung transplant with 1-year survival ranging between 47% and 76%, and 5-year survival ranging between 21% and 55%.^3^, ^4^, ^5^, ^6^ However, the studies reporting on these outcomes had great variability, composing both registry and single-center studies with varying local practices and patients undergoing ReTx procedures during the span of over 2 decades.

As primary lung transplant volume continues to increase, ReTx rates are also rising,^5^ thus there is growing interest in long-term survival and lung allograft outcomes as well as clinical factors affecting outcomes in the modern era. Further information is necessary to guide lung retransplant candidate selection and resource allocation to best optimize outcomes in this population. Given the considerable heterogeneity in the existing data, we sought to publish the ReTx outcomes at a high-volume, selective center with extensive medical and surgical expertise. We aimed to present our center’s data on ReTx over the last 2 decades and identify risk factors associated with poor outcomes.

## Methods

This was a retrospective analysis of patients who underwent lung transplantation at Columbia University Medical Center (CUMC). This study was approved by the Columbia University Institutional Review Board.

### Study design

We queried the United Network for Organ Sharing (UNOS) database for lung transplantations performed at our institution from May 4, 2005, which coincided with the implementation of the Lung Allocation Score (LAS) system, to October 31, 2023. Retrospective data collection was performed through the UNOS database and institutional electronic medical records. We included all patients who underwent primary lung transplantation and lung ReTx performed at our center. Exclusion criteria included recipient age less than 12 years, primary or ReTx procedures occurring at another institution, greater than one lung re-transplantation procedure, and multiorgan retransplantation.

### Statistical analysis

Descriptive statistics were generated for the included cohort. Baseline characteristics were compared between patients undergoing primary-only lung transplantation to those undergoing lung ReTx, using Kruskal-Wallis tests for continuous variables and chi-square for categorical variables. Survival rates following primary lung transplant and lung ReTx were analyzed using Cox proportional hazards regression models and were adjusted for age, type of transplant, and LAS. Freedom from CLAD for patients with lung ReTx was analyzed via log-rank test of Kaplan-Meier analysis. The primary outcomes were survival following transplant and freedom from CLAD. All data were analyzed using STATA, version 17 (Stata Corporation, College Station, Texas).

## Results

### Demographics

**Figure 1:**
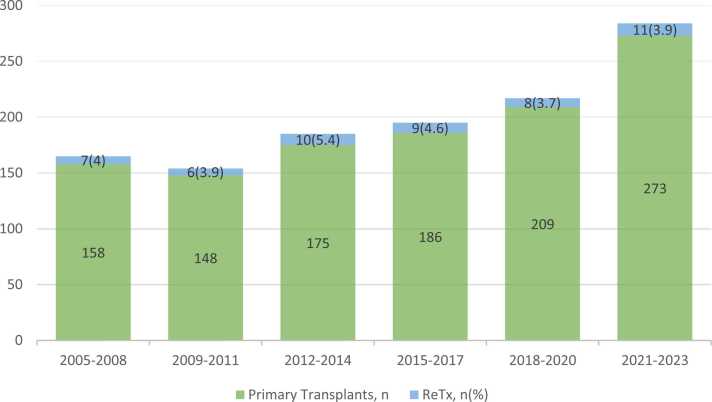
Number of lung transplants and retransplants performed at Columbia University Medical Center, 2005–2023.

Demographics are summarized in Table 1. Between May 5, 2005 and October 31, 2023, 1254 lung transplant procedures were performed at our institution. Of these, 52 patients (3.9%) underwent lung ReTx. The rate of ReTx over the study period is shown in Figure 1. One patient was excluded from the analysis for having undergone more than one lung ReTx procedure. Fifty-one patients with lung ReTx and 1149 patients with primary-only lung transplantation were included for final analysis with a median follow-up time of 7 years.

**Table 1:** Baseline characteristics

Characteristics		Primary Transplant (n = 1149)	ReTx (n = 51)	P-value
LAS, median		41 (36-47)	41 (37-47)	
Age (years), median		59 (49-64)	37 (31-58)	< 0.001
Sex	Male (%)	623 (54.2)	25 (49)	
	Female (%)	526 (45.8)	26 (51)	
Race/Ethnicity	Caucasian (%)	792 (68.9)	38 (74.5)	
	Black (%)	151 (13.1)	5 (9.8)	
	Hispanic (%)	145 (12.6)	6 (11.8)	
	Other (%)	61 (5.3)	2 (3.9)	
Surgical procedure	Single (%)	610 (53.1)	16 (31.4)	0.002
	Bilateral (%)	539 (46.9)	35 (68.6)	
Initial transplant indication	ILD (%)	739 (64.3)	17 (33)	< 0.001
	CF (%)	131 (11.4)	24 (47)	
	PH (%)	119 (10.4)	5 (10)	
	COPD (%)	160 (13.9)	5 (10)	
BMI, median		25 (22-29)	20 (18-25)	< 0.001
Pre-transplant ECMO (%)		85 (7.4)	3 (5.9)	
Pre-transplant MV (%)		53 (4.6)	10 (20)	< 0.001

Counts are presented as n (%); Medians are presented with 25th-75th quartile range

ReTx, retransplant; LAS, Lung Allocation Score; ILD, interstitial lung disease; CF, cystic fibrosis; PH, pulmonary hypertension; COPD, chronic obstructive pulmonary disease; BMI, body mass index; ECMO, extracorporeal membrane oxygenation; MV, mechanical ventilation

Retransplant patients had a median age of 37 years, were 51% female, predominantly Caucasian, and had a median BMI of 20. Most (69%) received bilateral lung ReTx. The median LAS at waitlisting was 41. A sizeable proportion of retransplant patients required mechanical ventilatory (MV) (20%) or extracorporeal membrane oxygenation (ECMO) (6%) bridge to ReTx.

Compared to primary-only lung transplant recipients, retransplant patients were overall younger (37 vs. 59 years, p <0.001), had lower BMI (20 vs. 25, p <0.001), were more likely to receive bilateral lung transplants (69% vs. 47%, p = 0.02), and more often required pre-transplant MV (20% vs. 4.6%, p <0.001). Retransplant patients most commonly underwent transplant initially for CF (47%), whereas most primary-only transplant recipients underwent transplant for ILD (64%) (p<0.001). LAS at listing, gender, ethnicity, and ECMO use were similar between groups.

### Characteristics and treatments in retransplant patients

The predominant indication for ReTx was CLAD-BOS (92%). Acute graft dysfunction was an uncommon indication for ReTx, accounting for only 4% of patients. The most common procedural sequence was bilateral lung transplantation for both the primary and ReTx operations (57%). Most ReTx occurred after 2 years following primary lung transplant (82%) with a median of 4.95 years (Table 2).

**Table 2:** Characteristics and treatments in ReTx recipients

Characteristics		N = 51
Indication for retransplant (%)	CLAD-BOS	47 (92)
	CLAD-RAS	2 (4)
	Acute graft dysfunction	2 (4)
Primary transplant procedure (%)	Single	13 (25)
	Bilateral	38 (75)
Retransplant procedure (%)	Single	16 (31)
	Bilateral	35 (69)
Transplant procedure sequence (%)	Single-Single	7 (14)
	Bilateral-Bilateral	29 (57)
	Single-Bilateral	6 (12)
	Bilateral-Single	9 (18)
Augmented immunosuppressive regimen during primary transplant course	Pulse dose steroids	36 (72)
	Pulse dose steroids and thymoglobulin	31 (62)
	Alemtuzumab	1 (2)
	Antibody-mediated rejection therapy	9 (18)
Induction agent (%)	Basiliximab	38 (81)
	Alemtuzumab	8 (17)
Interval between primary and ReTx (%)	90 days-1 year	5 (10)
	1-2 years	4 (8)
	2-5 years	17 (33)
	> 5 years	25 (49)
De novo DSA (%)	Primary transplant	17 (34)
	ReTx	8 (16)
Cause of death (%)	Graft failure	9 (31)
	Infection	11 (38)
	Cancer	3 (10)
	CVA	1 (3)
	Other	5 (17)

Counts are presented as n (%); Medians are presented with 25th-75th quartile range

ReTx, retransplant; CLAD-BOS, chronic lung allograft dysfunction, bronchiolitis obliterans; CLAD-RAS, chronic lung allograft dysfunction, restrictive allograft syndrome; DSA, donor specific antibody; CVA, cerebrovascular accident

Most patients (81%) received Basiliximab as an induction agent at ReTx with 17% receiving Alemtuzumab. Two percent of patients did not receive induction therapy at ReTx. Most patients were prescribed tacrolimus, mycophenolate, and prednisone as standard immunosuppressive agents after both primary and ReTx procedures. During the primary transplant course, most retransplant patients received at least one treatment of pulse dose steroids alone (72%) or the combination of pulse dose steroids and thymoglobulin (62%). Additionally, 18% of patients were treated with antibody-mediated rejection therapy with either plasmapheresis, rituximab, or carfilzomib. Thirty-four percent of patients developed de novo donor-specific antibody (DSA) after primary transplant (88% with class II HLA Ab), and 16% after ReTx (88% with class II HLA Ab) (Table 2).

### Primary outcomes

#### Survival

**Figure 2:**
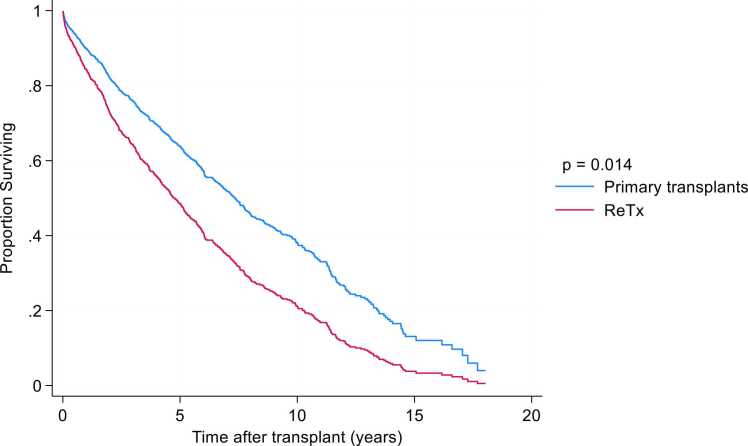
Survival in primary and ReTx recipients.

In our cohort, 1-, 3-, 5-, and 10-year survival rates following ReTx were 96%, 72%, 55%, and 28%, respectively (median = 5.61 [3.30–8.51] years). Corresponding survival rates after primary lung transplantation at our center were 88%, 74%, 63%, and 39% (median 7.34 [6.70–7.90] years). After adjusting for recipient age, type of surgical procedure, and LAS, survival following ReTx remained significantly lower compared with primary transplantation (adjusted HR 1.6; 95% CI, 1.10–2.35) (Figure 2). The leading causes of death after ReTx were infection (38%) and graft failure (31%) (Table 2).

**Figure 3:**
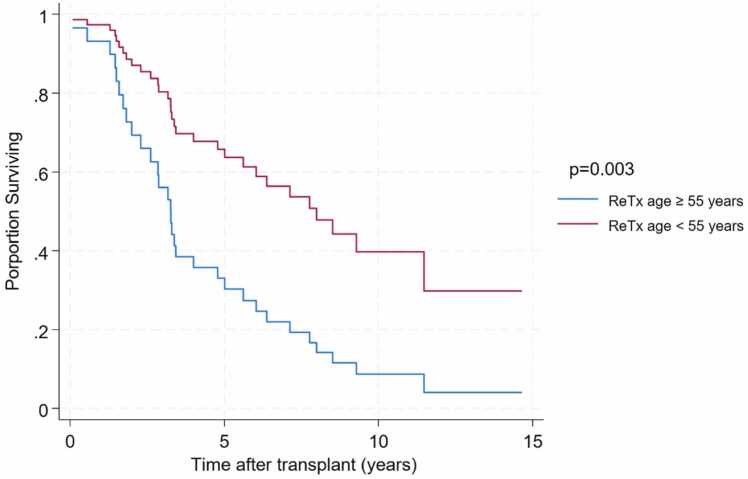
Survival in ReTx recipients stratified by age.

Recipient age ≥55 years at the time of ReTx was significantly associated with decreased survival (adjusted HR 2.6; 95% CI, 1.39–5.02) (Figure 3). In contrast, pretransplant MV or ECMO support, type of surgical procedure, and the interval between primary and re-transplantation were not associated with survival differences.

#### Lung allograft outcomes

**Figure 4:**
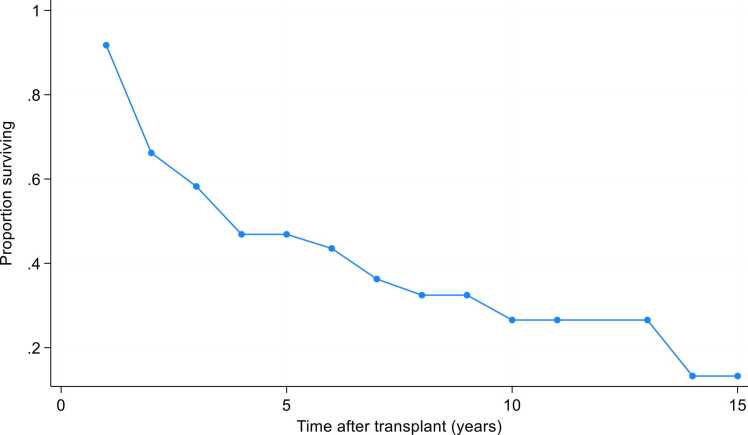
CLAD-free survival in ReTx recipients.

Freedom from CLAD stage 1 at 3 and 5 years after ReTx was 58% and 47%, respectively. Freedom from CLAD stage 2 at the same time points was 62% and 49%. The median CLAD-free survival within our cohort was 3.3 [1.7–7.3] years (Figure 4). Due to incomplete historical PFT data among primary-only lung transplant recipients, a direct comparison of CLAD-free survival between the ReTx and primary transplant cohorts could not be performed.

##### Association of early-onset CLAD development after ReTx and survival

**Figure 5:**
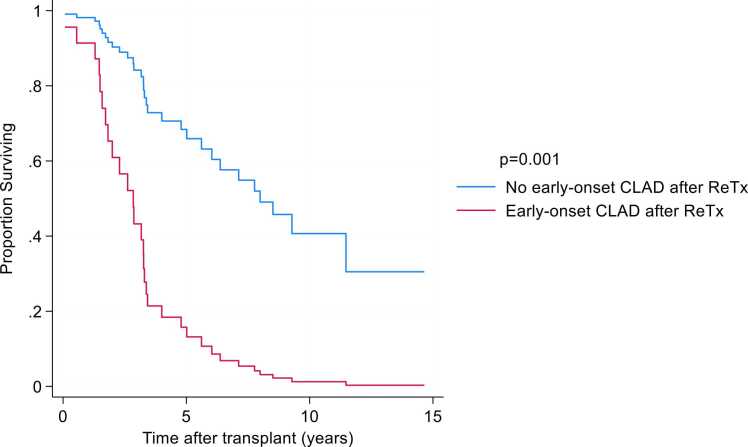
Survival in ReTx recipients stratified by early-onset CLAD within 2 years following ReTx.

Early-onset CLAD, defined as development of CLAD stage 1 within 2 years following ReTx, occurred in 34% of recipients. This subgroup demonstrated significantly worse survival compared with those without early-onset CLAD (HR 4.86; 95% CI, 1.94–12.2) (Figure 5). Three- and 5-year survival rates were 29% and 9%, respectively, among retransplant patients with early-onset CLAD, vs. 100% and 78% among those without. Three patients who died within 2 years of ReTx without available PFT data were excluded from analysis. The only significant difference in baseline or treatment characteristics between groups was a higher incidence of tacrolimus intolerance in the early-onset CLAD cohort (p = 0.0049). Tacrolimus intolerance was secondary to CNS complications in all patients: one patient in the early-onset CLAD group was switched to cyclosporine due to seizures while on tacrolimus, whereas the others developed altered mental status. Comparison between retransplant patients with and without early-onset CLAD is shown in Table S1.

##### Impact of early-onset CLAD development following primary transplant on CLAD-free survival after ReTx

We also examined whether the early-onset CLAD following the primary transplantation was associated with an increased risk of CLAD development after re-transplantation. Although there was a trend toward reduced CLAD-free survival following ReTx in the subset of early-onset CLAD developers from primary transplant, the association did not reach statistical significance.

## Discussion

Although post-ReTx survival at our center was significantly decreased compared to those following primary-only lung transplantation, our findings suggest more favorable outcomes than previously described. In this current large single-center retrospective study spanning nearly 2 decades (2005–2023), our aggregate ReTx survival at 1-, 3-, 5-, and 10-year was 96%, 72%, 55%, and 28%, respectively with median survival of 5.61 years.

Our study provides insight into the ReTx outcomes of a single, high-volume center with extensive experience. Compared to prior reports, our center appeared more selective in choosing candidates for ReTx. Despite performing over 1000 lung transplants during the 18-year study period, ReTx accounted for only 3.9% of our total volume, which was lower than the 4–16% reported in previous single-center studies across the U.S., North America, and Europe,^3^, ^6^, ^7^, ^8^, ^9^ and the 4–6% reported in the ISHLT registry between 2005 and 2017.^5^ Compared to the ISHLT registry data, our retransplant recipients were also younger (median age 37 years vs. 46 years) and had lower rates of ReTx for acute graft dysfunction (4% vs. 17%), and had longer interval between primary and ReTx procedures (median interval of 4.9 vs. 3.4 years).^5^ These factors had all been previously linked to ReTx survival differences.^3^, ^4^, ^5^, ^6^, ^7^, ^8^, ^10^, ^11^, ^12^, ^13^ This selective approach likely contributed to the superior survival outcomes observed. Furthermore, given our finding of reduced CLAD-free survival in retransplant patients with early-onset CLAD after primary transplant, our institutional practice of avoiding retransplantation in this subgroup may also have played a role in the favorable outcomes.

Similar to prior reports,^3^, ^5^, ^8^, ^14^ we observed that older recipient age was significantly associated with decreased survival following ReTx. However, unlike prior studies,^5^, ^8^, ^10^, ^11^, ^12^, ^15^ we did not find that pre-transplant MV or ECMO use was significantly associated with poorer outcomes, despite comparable rates of MV and ECMO use between our cohort and in the ISHLT registry. We also did not observe a significant impact of shorter interval between primary and retransplant procedures or of surgical procedure type on survival. However, the lack of association may be due to the characteristics of our cohort which had substantially longer interval between transplants and consisted mostly of bilateral transplant procedures for both primary and retransplantation, both of which characteristics were reported to be associated with improved survival.^3^, ^5^, ^6^, ^8^, ^10^, ^11^, ^12^

Our observed CLAD-free survival at 3 and 5 years following ReTx was comparable to prior reports.^8^, ^13^ It should be noted that although death was a competing event, due to the small sample size, we did not perform competing risk analysis. Within our cohort, 29% of ReTx recipients developed early-onset CLAD within 2 years after ReTx. This subgroup experienced markedly worse outcomes, with a 5-year survival rate of 9% compared to 78% among those without early-onset CLAD. The only factor that significantly differed between these groups was tacrolimus intolerance after both the primary and retransplant procedures. This finding is plausible, as cyclosporine-based immunosuppressive regimens have been shown to be inferior to tacrolimus-based therapy, with higher rates of acute cellular rejection and increased CLAD incidence.^16^, ^17^, ^18^, ^19^, ^20^ Future studies are warranted to validate this association, and caution is advised when considering ReTx in candidates with tacrolimus intolerance.

### Limitations

This was a single-center study, and local practice and preferences may not be representative of other centers. Specifically, the reported outcomes may not be generalizable to other centers with differing patient selection criteria and resource availability. Additionally, donor-specific characteristics were not included in the analysis. However, existing literature suggests that recipient characteristics are stronger predictors of post-ReTx survival outcomes.^11^, ^12^

## Conclusion

We report more optimistic single-center survival outcomes following lung ReTx. Our findings underscore the importance of careful candidate selection in optimizing post-ReTx survival. Future efforts to develop multicenter registries with prospective data collection are crucial for improving risk stratification and refining candidate selection criteria. Additionally, efforts to establish consensus guidelines for ReTx candidate selection should be considered.

## Declaration of Competing Interest

The authors declare that they have no known competing financial interests or personal relationships that could have appeared to influence the work reported in this paper.

## Footnotes

Appendix A Supplemental data associated with this article can be found in the online version at doi:10.1016/j.jhlto.2026.100501.
