# Supplementary material for: Lung retransplantation during the Lung Allocation Score era: Outcomes from a large single center
Source: JHLT Open. 2026 Jan 30;12:100501. doi: 10.1016/j.jhlto.2026.100501 (PMC12933294; doi:10.1016/j.jhlto.2026.100501)
Supplement: Supplementary file 1 — Supplemental material [file mmc1.docx]

**Lung retransplantation during the Lung Allocation Score era: Outcomes from a large single center**

Kemarut Laothamatas, MD (1); Cristiana Salvatori, MD (2); Harpreet Singh Grewal, MD (1); Mark Sonnick, MD (3); Elena-Rodica Vasilescu, MD (4); Lori Shah, MD (1); Hilary Robbins, MD (1); Angela DiMango, MD (1); Gabriela Magda, MD (1); Bryan P. Stanifer, MD, MPH (1,5); Joshua R. Sonett, MD (1,5); Frank D’Ovidio, MD, PhD (1,5) Philippe Lemaitre, MD, PhD (1,5); Luke Benvenuto, MD (1); Selim M. Arcasoy, MD, MPH (1)

1. Lung Transplant Program, Columbia University Irving Medical Center and New York-Presbyterian Hospital, New York, NY
2. Division of Pulmonary and Critical Care Medicine, Brigham and Women’s Hospital, Boston, MA
3. Lung Transplant Program, Transplant Institute, NYU Langone Health, New York, NY
4. Department of Pathology and Cell Biology, Columbia University Irving Medical Center and New York-Presbyterian Hospital, New York, NY
5. Section of Thoracic Surgery, Department of Surgery, Columbia University Irving Medical Center, New York, NY

**Supplementary Material**

**Table S1.** Characteristics of ReTx recipients with and without early-onset CLAD following ReTx

| Characteristics |  | No early-onset CLAD  (n = 33) | Early-onset ReTx CLAD  (n = 15) | p-value |
| --- | --- | --- | --- | --- |
| Age | | 36 (30-55) | 38 (34-60) |  |
| Sex | Male (%) | 17 (52) | 6 (40) |  |
|  | Female (%) | 16 (48) | 9 (60) |  |
| Race/Ethnicity | Caucasian (%) | 26 (79) | 10 (67) |  |
|  | Black (%) | 3 (9) | 2 (13) |  |
|  | Hispanic (%) | 2 (6) | 2 (13) |  |
|  | Other (%) | 2 (6) | 1 (7) |  |
| BMI | | 19 (16-26) | 19 (16-24) |  |
| Primary transplant Indication | ILD (%) | 3 (9) | 2 (13) |  |
|  | CF (%) | 5 (15) | 0 (0) |  |
|  | PH (%) | 16 (48) | 7 (47) |  |
|  | COPD (%) | 9 (27) | 6 (40) |  |
| ReTx Indication | CLAD-BOS (%) | 31 (94) | 15 (100) |  |
|  | Acute graft dysfunction (%) | 1 (3) | 0 (0) |  |
| Primary transplant procedure | Single (%) | 8 (24) | 3 (20) |  |
|  | Double (%) | 25 (76) | 12 (80) |  |
| ReTx procedure | Single (%) | 10 (30) | 5 (33) |  |
|  | Double (%) | 23 (70) | 10 (67) |  |
| Interval between primary and ReTx | 90 d-1 year (%) | 3 (9) | 0 (0) |  |
|  | 1-2 years (%) | 2 (6) | 2 (13) |  |
|  | 2-5 years (%) | 10 (30) | 7 (47) |  |
|  | > 5 years (%) | 18 (55) | 6 (40) |  |
| Pretransplant ECMO | | 1 (3) | 2 (13) |  |
| Pretransplant MV | | 5 (16) | 3 (20) |  |
| ReTx Induction | Basiliximab (%) | 26 (84) | 10 (77) |  |
|  | Campath (%) | 4 (13) | 3 (23) |  |
|  | Other/unknown (%) | 1 (3) | 0 (0) |  |
| Primary Transplant CNI^a^ | Tacrolimus (%) | 32 (97) | 12 (80) | 0.049 |
|  | Cyclosporine (%) | 1 (3) | 3 (20) |  |
| Primary transplant CCI | None (%) | 1 (3) | 0 (0) |  |
|  | Mycophenolate (%) | 26 (79) | 12 (80) |  |
|  | Azathioprine (%) | 6 (18) | 3 (20) |  |
| ReTx CNI^a^ | Tacrolimus (%) | 32 (97) | 12 (80) | 0.049 |
|  | Cyclosporine (%) | 1 (3) | 3 (20) |  |
| ReTx CCI | None (%) | 1 (3) | 2 (13) |  |
|  | Mycophenolate (%) | 30 (91) | 10 (67) |  |
|  | Azathioprine (%) | 2 (6) | 3 (20) |  |

**Table S1.** Characteristics of ReTx recipients with and without early-onset CLAD following ReTx

Counts are presented as n (%); Medians are presented with 25^th^-75^th^ quartile range

ReTx, retransplant; BMI, body mass index; CLAD, chronic lung allograft dysfunction; body mass index; ILD, interstitial lung disease; CF, cystic fibrosis; PH, pulmonary hypertension; COPD, chronic obstructive pulmonary disease; BOS, bronchiolitis obliterans; ECMO, extracorporeal membrane oxygenation; MV, mechanical ventilation; CNI, calcineurin inhibitor; CCI, cell cycle inhibitor

^a^One patient in the early-onset CLAD group was switched from tacrolimus to cyclosporine due to seizure. The remaining patients were switched to cyclosporine due to altered mental status.

**Figure S1.** Patient Flow Diagram

Patients undergoing lung transplantation at CUMC between May 4, 2005 to October 31, 2023 (n=1,254)

Excluded (n=1)

• >1 lung re-transplantation

1,149 Primary lung and 51 lung ReTx patients were included in the final analysis
